# cosinoRmixedeffects: an R package for mixed-effects cosinor models

**DOI:** 10.1186/s12859-021-04463-3

**Published:** 2021-11-13

**Authors:** Ruixue Hou, Lewis E. Tomalin, Mayte Suárez-Fariñas

**Affiliations:** 1 Department of Population Health Science and Policy, Icahn School of Medicine at Mount Sinai, New York, NY USA; 2 Department of Population Health Science and Policy, Mount Sinai Clinical Informatics Center, New York, NY USA; 3 Department of Genetics and Genomics, Icahn School of Medicine at Mount Sinai, New York, NY USA

**Keywords:** Circadian data, Cosinor, Mixed-effects, Wearable data, R package

## Abstract

**Background:**

Wearable devices enable monitoring and measurement of physiological parameters over a 24-h period, and some of which exhibit circadian rhythm characteristics. However, the currently available R package *cosinor* could only analyze daily cross-sectional data and compare the parameters between groups with two levels. To evaluate longitudinal changes in the circadian patterns, we need to extend the model to a mixed-effect model framework, allowing for random effects and interaction between COSINOR parameters and time-varying covariates.

**Results:**

We developed the *cosinoRmixedeffects* R package for modelling longitudinal periodic data using mixed-effects cosinor models. The model allows for covariates and interactions with the non-linear parameters MESOR, amplitude, and acrophase. To facilitate ease of use, the package utilizes the syntax and functions of the widely used *emmeans* package to obtain estimated marginal means and contrasts. Estimation and hypothesis testing involving the non-linear circadian parameters are carried out using bootstrapping. We illustrate the package functionality by modelling daily measurements of heart rate variability (HRV) collected among health care workers over several months. Differences in circadian patterns of HRV between genders, BMI, and during infection with SARS-CoV2 are evaluated to illustrate how to perform hypothesis testing.

**Conclusion:**

*cosinoRmixedeffects* package provides the model fitting, estimation and hypothesis testing for the mixed-effects COSINOR model, for the linear and non-linear circadian parameters MESOR, amplitude and acrophase. The model accommodates factors with any number of categories, as well as complex interactions with circadian parameters and categorical factors.

**Supplementary Information:**

The online version contains supplementary material available at 10.1186/s12859-021-04463-3.

## Background

Circadian rhythms play a key role in many physiological processes, including sleep/wake cycles, hormone secretion and neural function [1]. Wearable devices (such as smart watches) can monitor health-related physiological parameters and, coupled with AI technology, can provide low cost, non-invasive monitoring of chronic conditions [2], broadening access to health care. Many physiological parameters collected by wearable devices exhibit a circadian pattern (e.g., heart rate variability (HRV)). Current technology does not continuously record such parameters over a 24-h period, but rather follows a sparse and non-uniform sampling [3], therefore, readily derived variables (i.e. mean, range of the data within the day) would ignore this circadian pattern and introduce bias if used to develop AI algorithms, hindering accuracy. Therefore, development of techniques that appropriately model the non-uniform, sparsely sampled circadian rhythm data in a longitudinal setting, and implement a proper hypothesis testing framework, are needed to advance the use of integrated wearable data for prediction of any relevant outcomes.

Daily circadian rhythm has been modeled by a non-linear COSINOR method with three rhythm characteristics: the rhythm-adjusted mean (MESOR), half the extent of variation within a cycle (amplitude), and an angle relating to the time at which peak values recur in each cycle (acrophase) [4]. The currently available R package *cosinor* [5] implements such a model and allows the user to evaluate cross-sectional differences in the COSINOR parameters between groups with only two levels. However, if we have a daily circadian curve, repeated over a period of time, and we aim to investigate how this daily pattern changes in relationship to a factor or intervention, we cannot use the *cosinor* package, as it does not account for correlations within individuals. Therefore, to evaluate longitudinal changes in the circadian patterns, we extended the COSINOR model to a mixed-effect model framework, allowing for random effects and interaction between COSINOR parameters and time-varying covariates. We recently proposed a mixed-effect COSINOR model framework to evaluate changes in circadian HRV patterns after infection with SARS-CoV2 in healthcare workers [6], as well as to evaluate the association of HRV and self-reported stress [7].

Here, we present an open-source R package for our cosinor mixed-effect model, *cosinoRmixedeffects*, which can assume random circadian rhythm parameters for each subject when modelling longitudinal changes of daily data. *CosinoRmixedeffects* uses *lme4* package [8] for model fitting, and is integrated with the *emmeans* package [9] for obtaining estimated marginal means (EMMs) across levels of categorical factors (not only binary), and estimate differences between these levels. To illustrate utilization, *cosinoRmixedeffects* includes a vignette with detailed explanations of three models (Additional file 1). While the example framework in the vignette is described using HRV as an outcome, it can be applied to any outcome that displays circadian pattern characteristics. To the authors’ knowledge, *cosinoRmixedeffects* is the only R package currently available providing estimation and prediction of a mixed-effects COSINOR model for longitudinal periodic data with multi-level group comparisons.

## Implementation

The COSINOR model was used to model daily circadian rhythm over a 24 h period with the non-linear function:

1 $${\text{Y(t)}} = {\text{M}} + {\text{Acos}}\left( {{2}\uppi {\text{t}}/\uptau +\upphi } \right) + {\text{e}}_{{\text{i}}} ({\text{t}})$$

where τ, M, A and Φ are the period, MESOR, amplitude and acrophase respectively [4]. This non-linear model can be transformed into a linear model by recoding time (t) into two new variables x and z as x = cos(2πt/τ), z = sin(2πt/τ). HRV can then be written as:

2 $${\text{Y(t)}} = {\text{M}} +\upbeta \times {\text{x}}_{{\text{t}}} +\upgamma \times {\text{z}}_{{\text{t}}} + {\text{e}}_{{\text{i}}} {\text{(t)}}$$

To broaden this to a longitudinal framework, where individual circadian patterns change over a period of many days, we extended the model (Eq. 1) to a mixed-effect COSINOR model, where the measure Y of subject *i* at time *t* can be written as Y_it_ = (M + β × x_it_ + γ × z_it_) + W_it_ × θ_i_ + e_i_(t), e_i_(t) ~ N(0, s), where θ_i_ is a vector of random effects with multivariate normal distribution θ_i_ ~ N(0,Σ) that intrinsically modeled the within-patient correlation. To measure the impact of a covariate C on the daily circadian curve, we can include C as fixed effects and its interaction with x and z:

3 $${\text{Y}}_{{{\text{it}}}} = {\text{M}} + \upalpha _{{\text{o}}} {\text{C}}_{{\text{i}}} + \left( {\upbeta + \upalpha _{{2}} {\text{C}}_{{\text{i}}} } \right) \times {\text{x}}_{{{\text{it}}}} + \left( {\upgamma + \upalpha _{{3}} {\text{C}}_{{\text{i}}} } \right) \times {\text{z}}_{{{\text{it}}}} + {\text{W}}_{{{\text{it}}}} \times\uptheta _{{\text{i}}} + {\text{e}}_{{\text{i}}} ({\text{t}})$$

Hypothesis testing can be performed for any comparison, provided it can be written as a linear function of α’s, β and γ parameters.

To test for differences in COSINOR parameters M, A and ϕ between the populations defined by the covariate C, we used a bootstrapping procedure, where for each resampling iteration we:

1. Fit a linear mixed-effect model by reweighted least squares;
2. Estimate the marginal means obtaining the linear parameters for each group defined by C;
3. Use the inverse relationship to obtain EMMs for M, A and ϕ of each group defined by C;
4. Define the bootstrapping statistics as the pairwise differences of M, A and ϕ between groups defined by C.

These iterations were then used to define confidence intervals for the non-linear parameter with standard bootstrapping, and to derive *p*-values for the differences of each non-linear parameter, between groups defined by C.

In dealing with missing data, mixed-effects models utilize all observed data and produce robust estimates under the missing at random (MAR) assumption. On the other hand, generalized estimating equation (GEE) approaches and most imputation procedures, like complete case analysis and last-observed-carry-forward, require a much stronger missing completely at random (MCAR) assumption. As such, mixed-effects models are recommended as a more efficient and reliable method of primary analysis for clinical studies [10, 11]. If the number of missing values is large, the cosinor mixed-effects models could be followed up with sensitivity analysis, including imputation methods or combined with multiple imputation procedures.

## Results

The usage of *cosinoRmixedeffects* is illustrated with a subset of data from the Warrior Watch Study™ of Mount Sinai Health System (n = 121). In the study, health care workers were monitored by a smartphone app that remotely collected physiological data at non-uniform times across days [12]. HRV exhibits a 24-h circadian pattern, and changes in this pattern can be used to identify different physiological states [13]. HRV has also been found to be associated with infection and correlated with its severity [14]. Here, we present how to use the functions in the *consinoRmixedeffects* package to fit simple models comparing MESOR, amplitude and acrophase of HRV: (1) between COVID-19 positive and COVID-19 negative infection status and (2) across body mass index (BMI) categories within each biological sex.

A brief walkthrough of examples is outlined below, with details explained in Additional file 1. To estimate the effects of COVID-19 infection on HRV patterns, we can use a mixed-effects COSINOR model with sex and COVID-19 status as covariates and a random MESOR as:

HRV ~ sex + COVID + sex*x + sex*z + COVID*x + COVID*z + (1 | id)).

To model the data with *cosinormixedeffects* package, we follow 5 steps:

1. Use the *create.cosinor.param* function to create linear parameters:
  db<-create.cosinor.param(time="Hour",period=24,data=db)
2. Use the *fit.cosinor.mixed* function *to fit the desired model:*
  f<-fit.cosinor.mixed(y="hrv",x=c("sex","COVID"), random="1|id", data=db);
3. Estimate EMMs and 95% CI of MESOR, amplitude and acrophase by COVID-19 status using the function *get.means.ci.cosinor* defining *contrasts as they are defined* in the emmeans function
  db.m<-get.means.ci.cosinor(f, contrast.mean.frm="~COVID")
4. Use *ggplot.cosinor.lmer* to get the circadian curves by COVID-19 status (Fig. 1A),

**Fig. 1:**
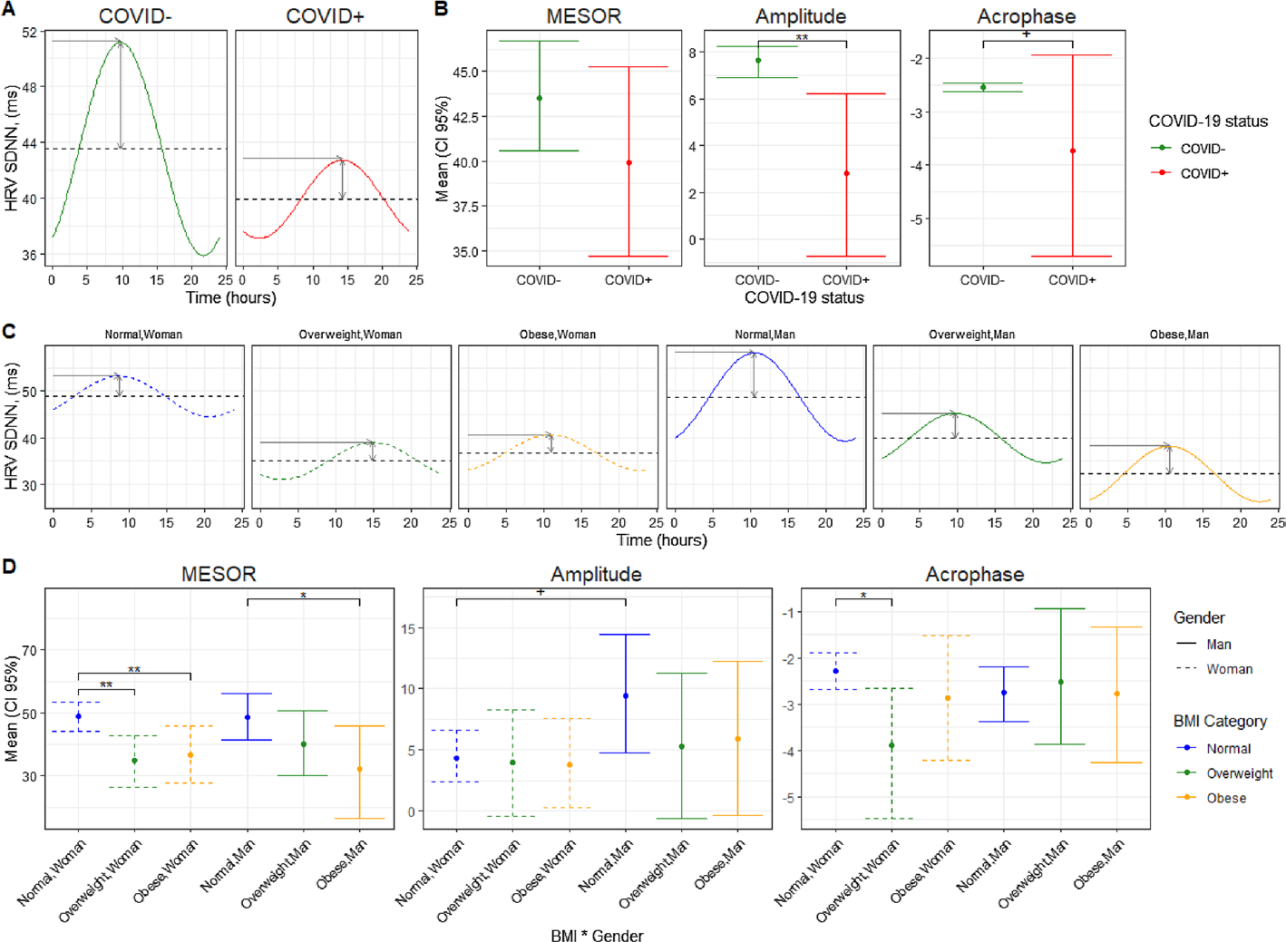
The association of COVID-19 status and BMI-sex interaction with HRV circadian rhythm. **A** Daily HRV pattern and **B** mean and 95% CI for the MESOR, Amplitude, and Acrophase for subjects with COVID-19 positive or negative status. **C** Daily HRV pattern and **D** mean and 95% CI for the MESOR, Amplitude, and Acrophase for each combination of biological sex by BMI category. Time (h) is indicated by the x-axis while HRV (ms) is indicated by the y-axis. Stars indicate significant differences, with (^+^*p* < 0.1; *p < 0.05; **p < 0.01; ***p < 0.001)
  p<-ggplot.cosinor.lmer(f,x_str="COVID",period=24,db.m, DATA=db)
  offers a core plot, which can be customized by adding more layers (p+labs()).
5. Use *get.contrasts.ci.cosinor* to test for differences in non-linear cosinor parameters by COVID-19 status using the bootstrap approach:
  db.delta<-get.contrasts.ci.cosinor(f,contrast.frm="~COVID");

To facilitate plotting the EMMs with significance of the contrasts, as depicted in Fig. 1B, the function *stat.test.stars()* adds the significance of the contrasts to the db.means data frame that can then be visualized with ggplot, exemplified in Additional file 1.

As shown in Fig. 1A, B, we found a statistically significant decrease in the amplitude and acrophase during SARS-CoV-2 infection, which is consistent with analysis showing that viral infection is associated with abnormal HRV [14].

Our package also allows the introduction of interaction terms to the COSINOR model. For example, to evaluate whether HRV rhythm is dependent on the relationship between BMI and biological sex, an interaction term sex*BMI_category can be added into the model and the means and contrasts can be estimated following the syntax of *emmeans.*

- f<-fit.cosinor.mixed(y="hrv", interaction=c("sex",
- "bmi_cat"), random="1+rrr+sss|id”, data=db.m)
- db.means<-get.means.ci.cosinor(fit=f,
- contrast.mean.frm="~bmi_cat|sex", nsim=500)
- db.delta<-get.contrasts.ci.cosinor(fit=f,
- contrast.frm="~bmi_cat|sex", nsim=500)

We found statistically significant differences in MESOR, amplitude and acrophase between groups (Fig. 1C, D). Sex differences in amplitude were observed only among subjects with normal BMI. A meta-analysis of 172 studies also revealed that HRV data obtained in men and women cannot be treated equally, potentially due to the sex differences in the autonomic control of the heart [15]. MESOR consistently decreased with BMI in both sexes, with obese and overweight women reaching their amplitude peak later than women with normal weight. Koenig et al. found that BMI was inversely associated with two time-related measures of HRV, and Yadav et al. also found differences in HRV parasympathetic and sympathetic indicators between obese and normal weight group [16, 17].

## Conclusions

*cosinoRmixedeffects* package provides functionality to model circadian rhythm outcomes in a longitudinal setting. Through a series of functions, the user can estimate the parameters of the COSINOR model, conduct hypothesis testing for the linear parameter and use bootstrap-based procedures to test hypothesis on the non-linear circadian parameters MESOR, amplitude and acrophase. The package accommodates factors in the model with any number of categories, and a variety of random effects, as well as complex interactions between circadian parameters and categorical factors. We expect that the availability of this package, will provide biomedical researchers with the proper tools to model circadian rhythm outcomes in a longitudinal setting, and also to be used as a circadian-conscious feature extraction tool for the development of AI algorithms for screening and monitoring health outcomes using wearable devices.

## Availability and requirements

- Project name: cosinoRmixedeffects
- Project home page: https://github.com/maytesuarezfarinas/cosinoRmixedeffects
- Operating system(s): Platform independent
- Programming language: R
- Other requirements: None
- License: GNU GPL v3
- Any restrictions to use by non-academics: No

## Supplementary Information

**Additional file 1. **Vignettes of package “cosinoRmixedeffects”.

## Data Availability

*cosinoRmixedeffects* is open-source and freely available from GitHub as an R package: https://github.com/maytesuarezfarinas/cosinoRmixedeffects; with example data available (the subset of Warrior Watch Study data used in examples) and vignettes included in the package.

## Abbreviations

HRV: Heart rate variability
BMI: Body mass index
MESOR: Rhythm-adjusted mean
