# Correction: cosinoRmixedeffects: an R package for mixed-effects cosinor models

**DOI:** 10.1186/s12859-022-04713-y

**Published:** 2022-05-17

**Authors:** Ruixue Hou, Lewis E. Tomalin, Mayte Suárez-Fariñas

**Affiliations:** 1 Department of Population Health Science and Policy, Icahn School of Medicine at Mount Sinai, New York, NY USA; 2 Department of Population Health Science and Policy, Mount Sinai Clinical Informatics Center, New York, NY USA; 3 Department of Genetics and Genomics, Icahn School of Medicine at Mount Sinai, New York, NY USA

## Correction to: Hou et al. BMC Bioinformatics (2021) 22:553 10.1186/s12859-021-04463-3

Following the publication of the original article [1], the authors would like to correct the sentence under the heading **Implementation**.

The sentences currently reads:

1. Fit a linear mixed-effect model by reweighted least squares;

The sentences should read:

1. Fit a linear mixed-effect model by restricted maximum likelihood;

The original article [1] has been corrected.
