# Supplementary material for: cosinoRmixedeffects: an R package for mixed-effects cosinor models
Source: BMC Bioinformatics. 2021 Nov 13;22:553. doi: 10.1186/s12859-021-04463-3 (PMC8590130; doi:10.1186/s12859-021-04463-3)
Supplement: Supplementary file 1 — Additional file 1. Vignettes of package “cosinoRmixedeffects”. [file 12859_2021_4463_MOESM1_ESM.pdf]

cosinoRmixedeffect: an R package for mixed-effects cosinor models

Ruixue Hou,Lewis E Tomalin, Mayte Suárez-Fariñas

02/2021

About

The *cosinoRmixedeffects* package can be implemented for the estimation and prediction of the a mixed-effects cosinor model for longitudinal periodic data. *cosinoRmixedeffects* package fits a mixed-effects cosinor model and utilizing the functionality of the emmeans package provides estimated marginal means (EMMs) and contrast for binary, categorical predictors and their interactions. In the example presented here, we will use a circadian rhythm data collected over several days to estimate daily changes assuming random intercept for each subject and a correlated covariance structure.

Cosinor models can be used to model daily circadian rhythm by estimating three components (see Cornélissen (2014) for details).

1. MESOR – a non-linear function that depends on the rhythm-adjusted mean
2. Amplitude - half the extent of variation within a day
3. Acrophase - the time of overall high values recurring in each day

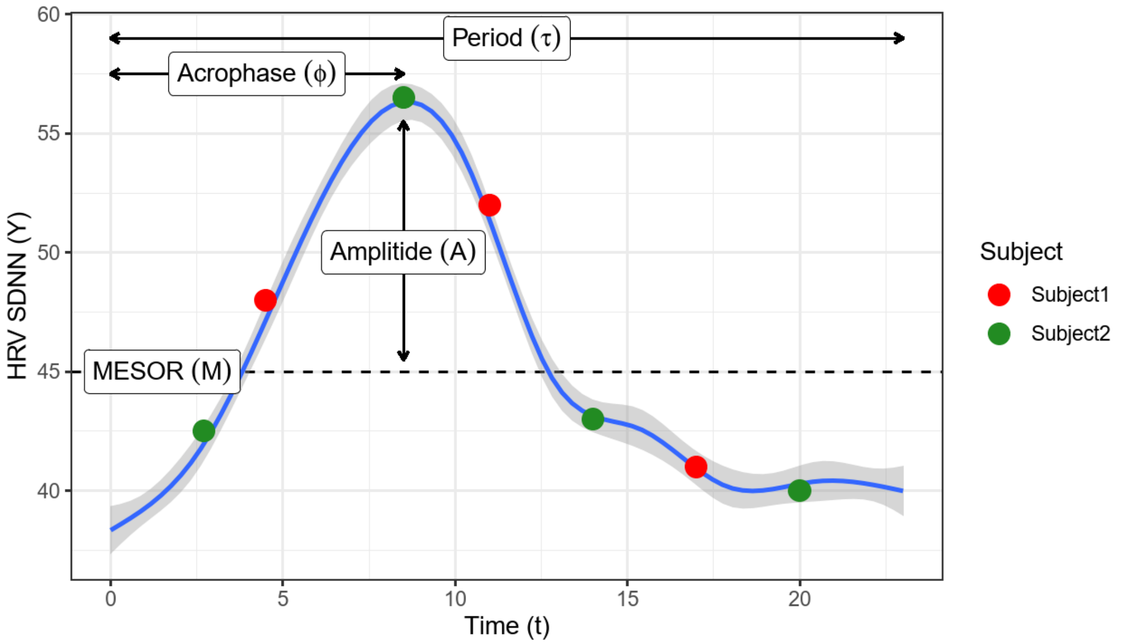

Red and green dots represent hypothetical sampling times though the day from two subjects with the same daily curve, showing that features like maximum, or range will be easily biased by the sampling time.

Currently, it is possible to model rhythmic data in R using a *cosinor* package for only daily cross-sectional designs, limited to simple group comparisons (i.e., 2-level factors). Hypothesis testing difference between groups with more than two levels or interactions are not allowed.Therefore, we extended the COSINOR model to a mixed-effects COSINOR model, to allow for longitudinal study designs where multiple predictors can be evaluated within a single model.

We will analyze a subset of the Warrior Watch Study data collected by the researchers at the Icahn School of Medicine at Mount Sinai. Health care workers (n=121) were monitored by a novel smartphone App employed remotely to collect biometric data, which included heart rate variability (HRV).HRV is physiological measure of time interval between heartbeats and exhibits a 24-hour circadian pattern, where changes in this pattern can be used to identify different physiological states, including infection onset (see Hirten.Robet.P, et al. for details about Warrior Watch Study).

cosinoRmixedeffects functionality for the Warrior Watch Study is available in the preprint:

1. Hirten, Robert P., et al. "Longitudinal Physiological Data from a Wearable Device Identifies SARS-CoV-2 Infection and Symptoms and Predicts COVID-19 Diagnosis." medRxiv (2020).

Installation

```
remotes::install_github("maytesuarezfarinas/cosinoRmixedeffects")

library(cosinoRmixedeffects)
```

Usage

Installation

```
remotes::install_github("maytesuarezfarinas/cosinoRmixedeffects")
library(cosinoRmixedeffects)
```

Load the package

```
require(emmeans)
require(lme4)
require(ggplot2)
require(reshape2)
require(dplyr)
require(limma)
```

Load the data

The dataset db.cosinor is the data with 6 variables.

1. participant_id is the ID of the participant
2. hrv is the heart rate variability
3. gender is the biological sex for the participants
4. T0tOT14 is COVID infection status within the first 14 days of COVID test

5. bmi_baseline_cat is the baseline BMI categories as normal weight, overweight and obese
6. Hour_of_Day is the time of the day when hrv data is measured

```
data(db.cosinor)

head(db.cosinor)
#>   participant_id      hrv gender  T0toT14 bmi_baseline_cat Hour_of_Day
#> 1            ID1  61.0108    Man   COVID-           Normal           2
#> 2            ID1  81.4739    Man   COVID-           Normal          21
#> 3            ID1  96.5104    Man   COVID-           Normal           1
#> 4            ID1  60.1389    Man   COVID-           Normal           2
#> 5            ID1  64.5900    Man   COVID-           Normal          17
#> 6            ID1 138.3790    Man   COVID-           Normal          17
```

Create cosinor parameters based on the time and cosinor period

This step calculates the rrr and sss for the cosinor model, with rrr=cos(2πt/τ),sss=sin(2πt/τ), where t is the time and τ is the period of a cycle. Here the time (t) is Hour of the day and the period (τ) is 24 hours.

```
## create cosinor paramenters needed for cosinor model
db.model<-create.cosinor.param(time="Hour_of_Day", period=24,
data=db.cosinor)
```

Examine the data

```
## the number of participants with COVID+ and COVID-
table(db.model[!duplicated(db.model$participant_id), c("T0toT14")])
#>
#> COVID- COVID+
#>    111     10

## the number of male and female participants
table(db.model[!duplicated(db.model$participant_id), c("gender")])
#>
#> Woman   Man
#>    84    35

## the number of normal weight, overweight and obese participants
table(db.model[!duplicated(db.model$participant_id), c("bmi_baseline_cat")])
#>
#>     Normal Overweight      Obese
#>         65         28         20
```

Using these data, we will provide three modeling examples with *cosinoRmixedeffects*'s functionality:

1. Model 1 that fits a model for biological sex (Female vs. Male), assuming random effects for MESOR;

2. Model 2 that fits COVID-19 status (positive vs negative) and adjusts for biological sex as a covariate, assuming random effects for MESOR;
3. Model 3 that fits an interaction between biological sex and BMI, assuming random effects for MESOR, amplitude and acrophase.

Example 1. Fit model for biological sex only, assuming random effects for MESOR

Step 1. fit mixed-effects cosinor model to estimate the effects of biological sex on HRV curves (MESOR, amplitude and acrophase) using *fit.cosinor.mixed()* function.

The arguments "y", "random" and "data" are required, and inputs for either argument "x" or "interaction" are needed. Here, we only assumed random effects for MENSOR (random intercept) in this example.We could also assume random effects for MENSOR, amplitude and acrophase by changing the argument *random="1|participant_id"* to *random = "1+rrr+sss|participant_id"*. The default action to handle missing data is "na.action=na.omit", where it returns the object with observations removed if they contain any missing values.

```
## Fit Model 1
f1<-fit.cosinor.mixed(y="hrv",x="gender",random="1|participant_id",
data=db.model)
```

Step 2. get the estimated mean MESOR, amplitude and acrophase by gender, and use bootstrapping to get confidence intervals for the means,with function *get.means.ci.cosinor*.

This function uses the inverse relationship to obtain EMMs for MESOR, amplitude and acrophase.The model fit object and contrast formula are required for this function. The default number of simulations is 500. The more simulations we conduct, the closer distribution/probability we get to the theoretical distribution/probability.The default is to calculate 95% confidence interval, but argument *conflevel* could be specified to obtain other confidence intervals, e.g. conflevel = 0.9 for 90% confidence interval . The type of interval calculated by default is "norm", but other types are also allowed by specifying the argument *conftype*.

```
db.means<-get.means.ci.cosinor(f1, contrast.frm="~gender",nsim=500)
db.means
#>        MEAN     2.5 %    97.5 %           VALUE     Param gender
#> 1 43.775624 40.673599 46.771250     MESOR_Woman     MESOR  Woman
#> 2 42.902322 38.038963 48.360760       MESOR_Man     MESOR    Man
#> 3  5.210668  4.575937  5.980992 Amplitude_Woman Amplitude  Woman
#> 4  9.808858  8.564749 11.248533   Amplitude_Man Amplitude    Man
#> 5 -2.660304 -2.795657 -2.510189 Acrophase_Woman Acrophase  Woman
#> 6 -2.521834 -2.629722 -2.421000   Acrophase_Man Acrophase    Man

## the MEAN is the EMM, with 95% confidence interval lower boundary as "2.5%"
and upper boundary as "97.5%" columns.
```

Step 3. get the estimated pairwise contrasts by gender and confidence intervals for MESOR, Amplitude and acrophase of hrv, with function *get.contrasts.ci.cosinor*.

The model object and contrast formula are required for this function.The default is 95% confidence interval, but argument *conflevel* could be specified to obtain other confidence intervals, e.g. conflevel = 0.9 for 90% confidence interval . The type of interval calculated by default is “norm”, but other types are also allowed by specifying the argument *conftype*.

```
db.delta<-get.contrasts.ci.cosinor(f1,contrast.frm="~gender",nsim=500)
db.delta
#>                                           2.5 %    97.5 % boot.estimate
boot.SE
#> MESOR_Woman_vs_MESOR_Man         -6.91082561 4.9470875    -0.8733013
3.0250334
#> Amplitude_Woman_vs_Amplitude_Man  3.11133908 6.1369973     4.5981901
0.7718658
#> Acrophase_Woman_vs_Acrophase_Man -0.01927069 0.2974999     0.1384693
0.0808103
#>                                  pvalue
#> MESOR_Woman_vs_MESOR_Man           0.78
#> Amplitude_Woman_vs_Amplitude_Man   0.00
#> Acrophase_Woman_vs_Acrophase_Man   0.12

## boot.estimate is the estimated contrasts between groups, with its 95%
confidence interval lower boundary as "2.5%" and upper boundary as "97.5%"
columns.
```

Step 4. plot mean and CI for COSINOR parameters.

```
## This uses the db.means object you created earlier
p<-ggplot(db.means, aes(x=gender, y=MEAN, color=gender))+
  geom_point(position = position_dodge(width=1))+
  scale_color_manual(values = c(
    Woman="forestgreen",
    Man="orange"))+
  geom_errorbar(aes(ymin=`2.5 %`, ymax=`97.5 %`),position =
position_dodge(width=1))+
  theme_bw()+
  facet_wrap(~Param, scales = "free_y") +
  guides(color=guide_legend(title="Gender"))+
  labs(x="Gender", y="Mean (CI 95%)") +
  theme(strip.background = element_rect(color="black", fill="white",
linetype=0))+
  theme(strip.text.x = element_text(size = 14))
```

Here we used the function *{stat.test.stars}* to obtain stars to indicate statistical significance levels, and this function requires db.delta and contrast formula.

```
require(rstatix)
stat.test<-stat.test.stars(db.delta=db.delta, contrast.frm = "~gender")

require(ggpubr)
p<-p+stat_pvalue_manual(stat.test, hide.ns = T, step.increase = 0.05,
```

```
tip.length = 0.03)

p
```

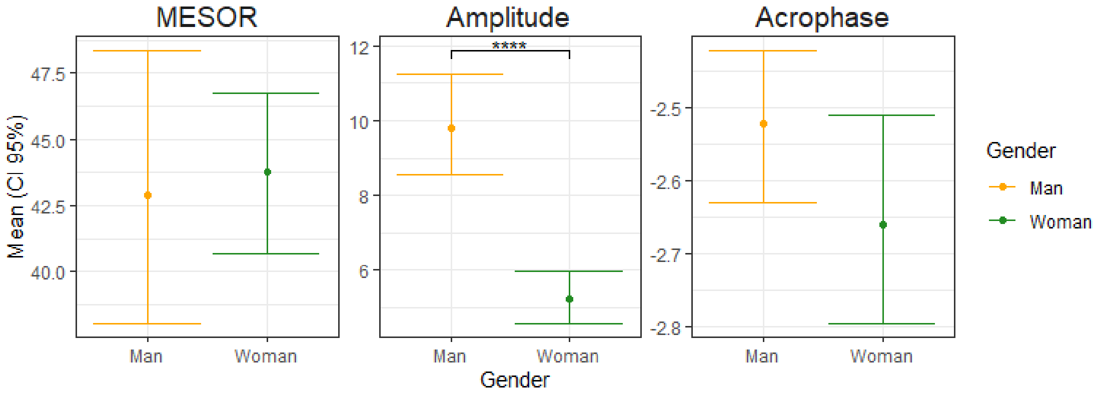

Step 5. plot the COSINOR circadian curves with the function *ggplot.cosinor.lmer*.

We need the fit object, the group variable, the period of a cycle, the time variable

```
## ## This code creates blank axis, and relies on model fit “f1”, estimated
means “db.means”, and original data “db.model” objects.
p<-ggplot.cosinor.lmer(object=f1,
                       x_str="gender",
                       period=24,
                       db.means=db.means,
                       DATA=db.model)

## This code customizes the plot
p + labs(x="Time (hours)", y="HRV SDNN, (ms)")+
  scale_color_manual(values=c(Man="orange", Woman="forestgreen"))+
  geom_hline(aes(yintercept=MESOR), linetype=2, color="black")+
  theme_bw()+
  facet_wrap(~gender, nrow = 1)+
  guides(color=guide_legend(title="Gender"))+
  geom_segment(aes(x = T_AMP,
                   y = MESOR+0.05,
                   xend = T_AMP,
                   yend = MESOR+Amplitude-0.05),
               arrow = arrow(length = unit(0.15, "cm"),ends="both"),
               size=0.5, color="gray50",linetype=1)+
  geom_segment(aes(x = 0,
                   y =  MESOR+Amplitude+0.1,
                   xend = T_AMP,
                   yend = MESOR+Amplitude+0.1),
               arrow = arrow(length = unit(0.15, "cm")), size=0.5,
color="gray50",linetype=1)+
  geom_line(aes(color=factor(GROUP, levels = c("Man", "Woman"))))
```

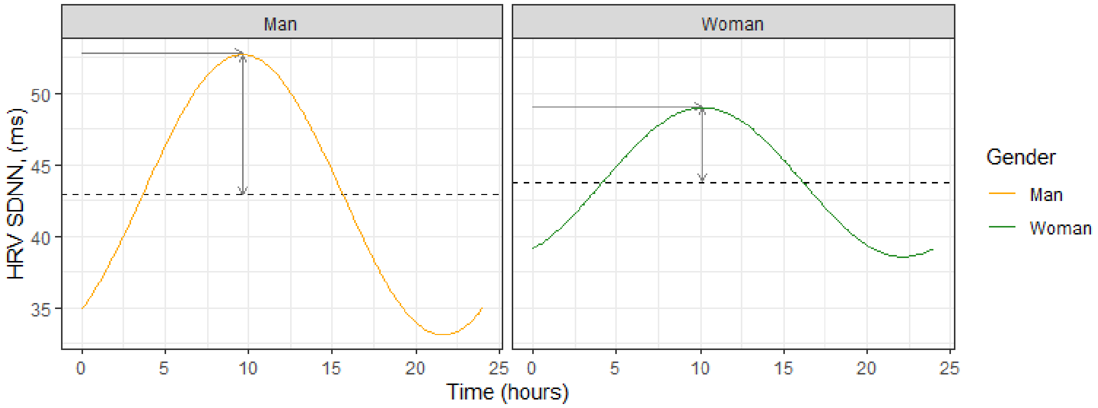

Example 2. Fit model to estimate effect of COVID-19 status on MESOR, Amplitude and Acrophase, adjusted for gender, assuming random effects for MESOR

Step 1. fit mixed effects cosinor model to estimate effect of COVID-19 status on MESOR, Amplitude and Acrophase, adjusted for gender.

This is achieved by adding both gender and T0toT14 variable in the argument "x" of the function *fit.cosinor.mixed*. More covariates could be added by adding more elements into the vector of argument "x".

```
f2<-
fit.cosinor.mixed(y="hrv",x=c("gender","T0toT14"),random="1|participant_id",
data=db.model)
```

Step 2. get the estimated transformed CONSINOR mean MESOR, amplitude and acrophase by COVID-19 status, adjusted for gender, and use bootstrapping to get confidence intervals for the means

```
db.means<-get.means.ci.cosinor(fit=f2, contrast.frm="~T0toT14", nsim=500)
db.means
#>        MEAN      2.5 %    97.5 %             VALUE     Param T0toT14
#> 1 43.525184 40.6726923 46.320606      MESOR_COVID-     MESOR  COVID-
#> 2 39.927396 35.1496417 44.961061      MESOR_COVID+     MESOR  COVID+
#> 3  7.647612  6.9662819  8.499839 Amplitude_COVID- Amplitude  COVID-
#> 4  2.807849 -0.7030568  6.359330 Amplitude_COVID+ Amplitude  COVID+
#> 5 -2.553112 -2.6434046 -2.475285 Acrophase_COVID- Acrophase  COVID-
#> 6 -3.733294 -5.3763713 -2.064178 Acrophase_COVID+ Acrophase  COVID+
```

This is to show EMMs by groups of biological sex, adjusted for COVID-19 status, and estimates changed compared to Model 1 with no covariates adjusted.

```
db.means2<-get.means.ci.cosinor(fit=f2, contrast.frm="~gender", nsim=500)
db.means2
#>        MEAN      2.5 %    97.5 %            VALUE     Param gender
#> 1 42.180969 38.8314759 45.263616      MESOR_Woman     MESOR  Woman
#> 2 41.271611 35.4276027 46.786228        MESOR_Man     MESOR    Man
```

```
#> 3  2.884162  0.5348876  5.014467 Amplitude_Woman Amplitude   Woman
#> 4  7.571298  5.3228833  9.805592   Amplitude_Man Amplitude     Man
#> 5 -3.368466 -4.1118164 -2.564947 Acrophase_Woman Acrophase   Woman
#> 6 -2.667759 -2.9339119 -2.391904   Acrophase_Man Acrophase     Man
```

Step 3. get the estimated pairwise contrasts by COVID-19 status and confidence intervals for MESOR, Amplitude and acrophase

```
db.delta<-get.contrasts.ci.cosinor(fit=f2,contrast.frm="~T0toT14", nsim=500)
db.delta
#>                                           2.5 %     97.5 % boot.estimate
#> MESOR_COVID-_vs_MESOR_COVID+         -8.074023  1.2114418     -3.597788
#> Amplitude_COVID-_vs_Amplitude_COVID+ -8.823540 -1.3528611     -4.839763
#> Acrophase_COVID-_vs_Acrophase_COVID+ -2.791219  0.2912272     -1.180182
#>                                         boot.SE pvalue
#> MESOR_COVID-_vs_MESOR_COVID+         2.3687846   0.17
#> Amplitude_COVID-_vs_Amplitude_COVID+ 1.9058203   0.01
#> Acrophase_COVID-_vs_Acrophase_COVID+ 0.7863527   0.12
```

Step 4. plot mean and CI for COSINOR parameters

```
p<-ggplot(db.means, aes(x=T0toT14, y=MEAN, color=T0toT14))+
  geom_point(position = position_dodge(width=1))+
  scale_color_manual(values = c(`COVID-`="forestgreen", `COVID+`="red"))+
  geom_errorbar(aes(ymin=`2.5 %`, ymax=`97.5 %`),position =
position_dodge(width=1))+
  theme_bw()+
  facet_wrap(~Param, scales = "free_y") +
  guides(color=guide_legend(title="COVID-19 status"))+
  labs(x="COVID-19 status", y="Mean (CI 95%)") +
  theme(strip.background = element_rect(color="black", fill="white",
linetype=0))+
  theme(strip.text.x = element_text(size = 14))

## The code below create the object stat.test, so you can add stars to the
plot and it requires db.delta
require(rstatix)
stat.test<-stat.test.stars(db.delta=db.delta, contrast.frm = "~T0toT14")

require(ggpubr)

p<-p+stat_pvalue_manual(stat.test, hide.ns = T, step.increase = 0.05,
tip.length = 0.03)

p
```

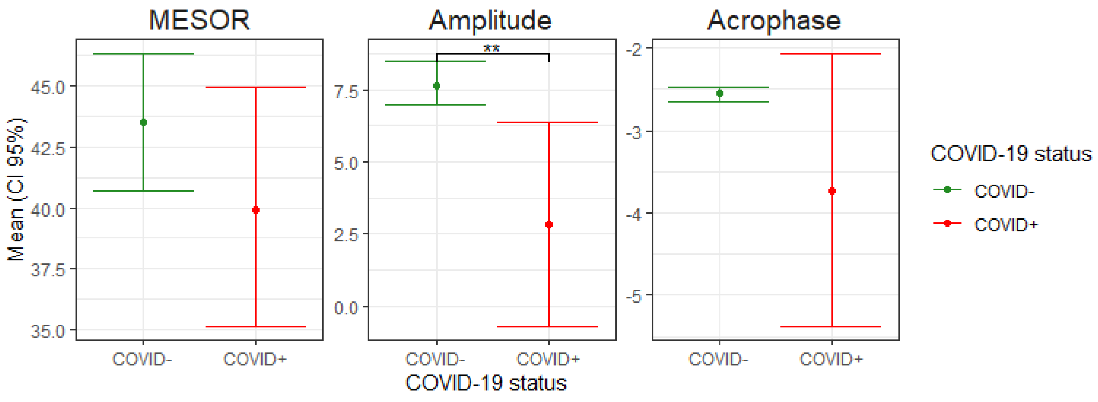

Step 5. plot the COSINOR circadian curves

```
## This code creates blank axis
p<-ggplot.cosinor.lmer(object=f2,
                       x_str="T0toT14",
                       period=24,
                       db.means=db.means,
                       DATA=db.model)

## This code customizes the plot
p + labs(x="Time (hours)", y="HRV SDNN, (ms)")+
  scale_color_manual(values=c(`COVID-`="forestgreen", `COVID+`="red"))+
  geom_hline(aes(yintercept=MESOR), linetype=2, color="black")+
  theme_bw()+
  facet_wrap(~T0toT14, nrow = 1)+
  guides(color=guide_legend(title="COVID-19 status"))+
  geom_segment(aes(x = T_AMP,
                   y = MESOR+0.05,
                   xend = T_AMP,
                   yend = MESOR+Amplitude-0.05),
               arrow = arrow(length = unit(0.15, "cm"),ends="both"),
               size=0.5, color="gray50",linetype=1)+
  geom_segment(aes(x = 0,
                   y =  MESOR+Amplitude+0.1,
                   xend = T_AMP,
                   yend = MESOR+Amplitude+0.1),
               arrow = arrow(length = unit(0.15, "cm")), size=0.5,
color="gray50",linetype=1)+
  geom_line(aes(color=factor(GROUP, levels = c("COVID-",
                                               "COVID+"))))
```

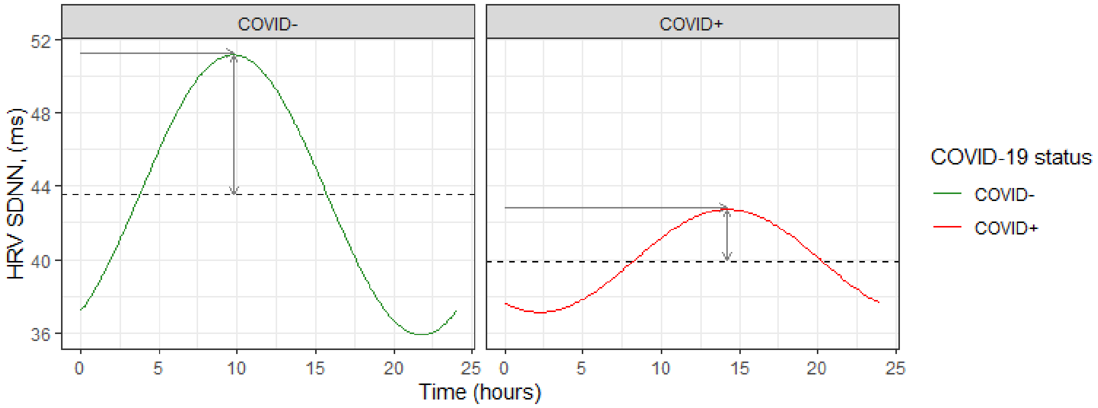

Example 3. Interaction model, interaction between gender and BMI category, assuming random effects for MESOR, amplitude and acrophase

Step 1. fit mixed effects cosinor model with interaction between gender and BMI category

This is achieved by adding variables of interaction terms into the argument "interaction" of the function *fit.cosinor.mixed*. The argument "x" could also be used to add other covariates in addition to interactions.

```
f3<-fit.cosinor.mixed(y="hrv",
                        interaction=c("gender","bmi_baseline_cat"),
                        random="1+rrr+sss|participant_id",
                        data=db.model)
```

Step 2. get the estimated transformed CONSINOR mean MESOR, amplitude and acrophase by BMI, after adjusting for gender, and use bootstrapping to get confidence intervals for the means

To get the estimated means across body mass index (BMI) categories within each sex, the contrast formula "~bmi_baseline_cat|gender" is written in the same syntax as *emmeans*.

```
db.means<-get.means.ci.cosinor(fit=f3,
contrast.frm="~bmi_baseline_cat|gender", nsim=500)
head(db.means)
#>        MEAN     2.5 %    97.5 %                  VALUE Param
#> 1 48.86768 44.72811 52.89942      MESOR_Normal,Woman MESOR
#> 2 34.98104 26.19773 43.97240 MESOR_Overweight,Woman MESOR
#> 3 36.74116 28.61308 45.54457       MESOR_Obese,Woman MESOR
#> 4 48.70818 40.48614 57.42145        MESOR_Normal,Man MESOR
#> 5 39.91667 30.92263 48.54916   MESOR_Overweight,Man MESOR
#> 6 32.25947 20.64921 44.34193         MESOR_Obese,Man MESOR
#>   bmi_baseline_cat_gender
#> 1            Normal,Woman
#> 2        Overweight,Woman
#> 3             Obese,Woman
#> 4              Normal,Man
```

```
#> 5          Overweight,Man
#> 6               Obese,Man
```

Step 3. get the estimated pairwise contrasts and the confidence intervals for MESOR, Amp, Acro for each BMI category

```
db.delta<-
get.contrasts.ci.cosinor(fit=f3,contrast.frm="~bmi_baseline_cat|gender",
nsim=500)
head(db.delta)
#>                                                    2.5 %    97.5 %
boot.estimate
#> MESOR_Normal,Woman_vs_MESOR_Overweight,Woman -22.30503 -4.300281   -
13.8866383
#> MESOR_Normal,Woman_vs_MESOR_Obese,Woman      -21.37807 -2.892373   -
12.1265216
#> MESOR_Normal,Woman_vs_MESOR_Normal,Man       -10.67278  9.399982    -
0.1595046
#> MESOR_Normal,Woman_vs_MESOR_Overweight,Man   -19.16185  1.245528    -
8.9510156
#> MESOR_Normal,Woman_vs_MESOR_Obese,Man        -31.46100 -1.302549   -
16.6082162
#> MESOR_Overweight,Woman_vs_MESOR_Obese,Woman  -10.50539 12.840260
1.7601166
#>                                              boot.SE pvalue
#> MESOR_Normal,Woman_vs_MESOR_Overweight,Woman 4.593132   0.01
#> MESOR_Normal,Woman_vs_MESOR_Obese,Woman      4.715824   0.02
#> MESOR_Normal,Woman_vs_MESOR_Normal,Man       5.120697   0.97
#> MESOR_Normal,Woman_vs_MESOR_Overweight,Man   5.206058   0.07
#> MESOR_Normal,Woman_vs_MESOR_Obese,Man        7.693623   0.01
#> MESOR_Overweight,Woman_vs_MESOR_Obese,Woman  5.955632   0.80
```

Step 4. plot mean and CI for COSINOR parameters

```


db.means<-mutate(db.means,
                 bmi_baseline_cat=strsplit2(bmi_baseline_cat_gender,",")[,1],
                 gender=strsplit2(bmi_baseline_cat_gender,",")[,2])



db.means<-mutate(db.means,
bmi_baseline_cat_gender=factor(bmi_baseline_cat_gender,
                                  levels = c("Normal,Woman",
"Overweight,Woman", "Obese,Woman" ,
                                             "Normal,Man" ,
"Overweight,Man", "Obese,Man")),
                 bmi_baseline_cat=factor(bmi_baseline_cat,
                                  levels = c("Normal", "Overweight",
"Obese")))
```

```r
p<-ggplot(db.means, aes(x=bmi_baseline_cat_gender, y=MEAN,
color=bmi_baseline_cat, linetype=gender))+
  geom_point(position = position_dodge(width=1))+
  scale_color_manual(values = c(
    "blue",
    "forestgreen",
    "orange"))+
  scale_linetype_manual(values=c("solid","dashed"))+
  geom_errorbar(aes(ymin=`2.5 %`, ymax=`97.5 %`),position =
position_dodge(width=1))+
  theme_bw()+
  facet_wrap(~Param, scales = "free_y") +
  guides(color=guide_legend(title="BMI Category"),
linetype=guide_legend(title="Gender"))+
  labs(x="BMI * Gender", y="Mean (CI 95%)") +
  theme(strip.background = element_rect(color="black", fill="white",
linetype=0))+
  theme(strip.text.x = element_text(size = 14),
axis.text.x=element_text(angle=45,hjust=1))


## The code below create the object stat.test, so you can add stars to the
plot and it requires db.delta
require(rstatix)
stat.test<-stat.test.stars(db.delta=db.delta, contrast.frm =
"~bmi_baseline_cat|gender")

require(ggpubr)

## comparisons of interests
comparisons<-c("Normal,Woman_vs_Overweight,Woman" ,
"Normal,Woman_vs_Obese,Woman" , "Overweight,Woman_vs_Obese,Woman" ,
                "Normal,Man_vs_Overweight,Man"   ,
"Normal,Man_vs_Obese,Man","Overweight,Man_vs_Obese,Man",
                "Normal,Woman_vs_Normal,Man" ,
"Overweight,Woman_vs_Overweight,Man","Obese,Woman_vs_Obese,Man")

stat.stars<-subset(stat.test, stat.test$contrast %in% comparisons)

p<-p+stat_pvalue_manual(stat.stars, hide.ns = T, step.increase = 0.05,
tip.length = 0.03, step.group.by="Param")

p
```

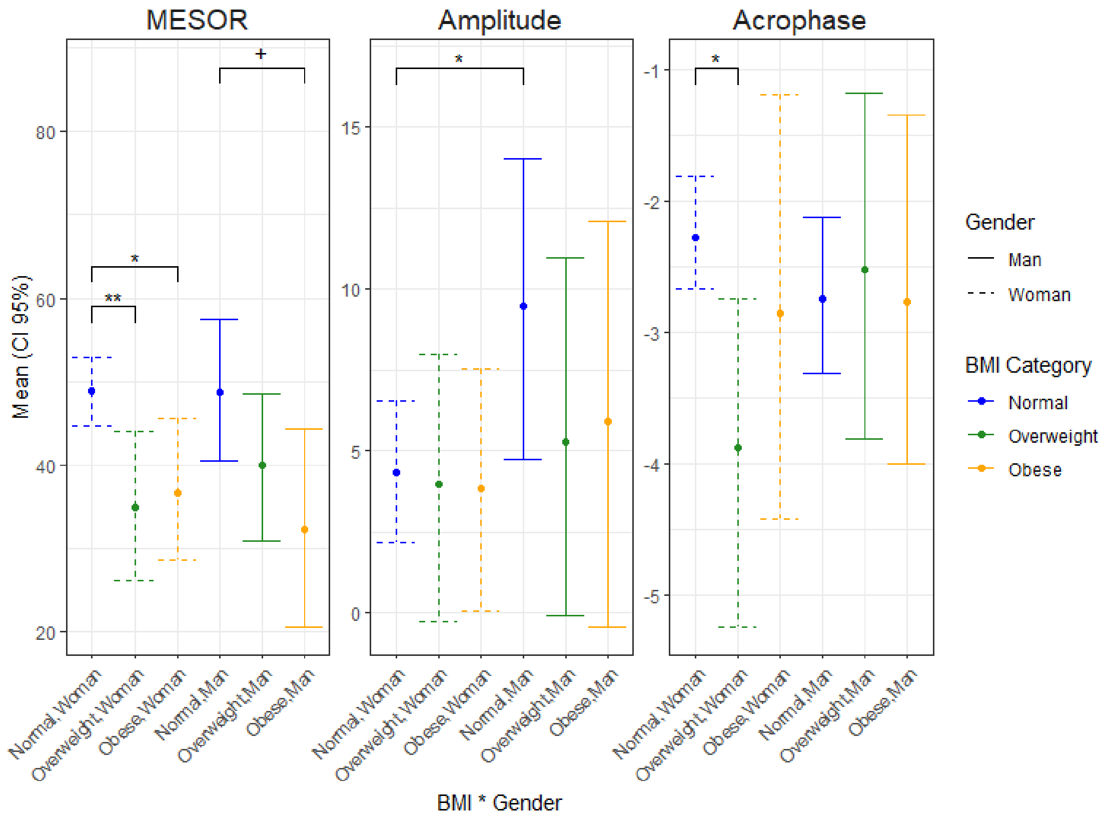

Step 5. plot the COSINOR circadian curves

```
## This code creates blank axis
p2<-ggplot.cosinor.lmer(object=f3,
                       x_str="bmi_baseline_cat_gender",
                       period=24,
                       db.means=db.means,
                       DATA=db.model)

## This code customizes the plots
p2<-p2 + labs(x="Time (hours)", y="HRV SDNN, (ms)")+
  scale_color_manual(values=c(
    "blue",
    "forestgreen",
    "orange",
    "red",
    "purple",
    "deepskyblue"))+
  geom_hline(aes(yintercept=MESOR), linetype=2, color="black")+
  theme_bw()+
  facet_wrap(~bmi_baseline_cat_gender, nrow = 1)+
  guides(color=guide_legend(title="BMI * Gender Category"))+
  geom_segment(aes(x = T_AMP,
                   y = MESOR+0.05,
                   xend = T_AMP,
```

```
                        yend = MESOR+Amplitude-0.05),
                    arrow = arrow(length = unit(0.15, "cm"),ends="both"),
                    size=0.5, color="gray50",linetype=1)+
  geom_segment(aes(x = 0,
                        y =  MESOR+Amplitude+0.1,
                        xend = T_AMP,
                        yend = MESOR+Amplitude+0.1),
                    arrow = arrow(length = unit(0.15, "cm")), size=0.5,
color="gray50",linetype=1)+
  geom_line(aes(color=factor(GROUP, levels =
c("Normal,Woman","Overweight,Woman","Obese,Woman",

"Normal,Man","Overweight,Man","Obese,Man"))))+
  theme(strip.background = element_rect(color="black", fill="white",
linetype=0))+
  theme(strip.text.x = element_text(size = 8))
p2
```

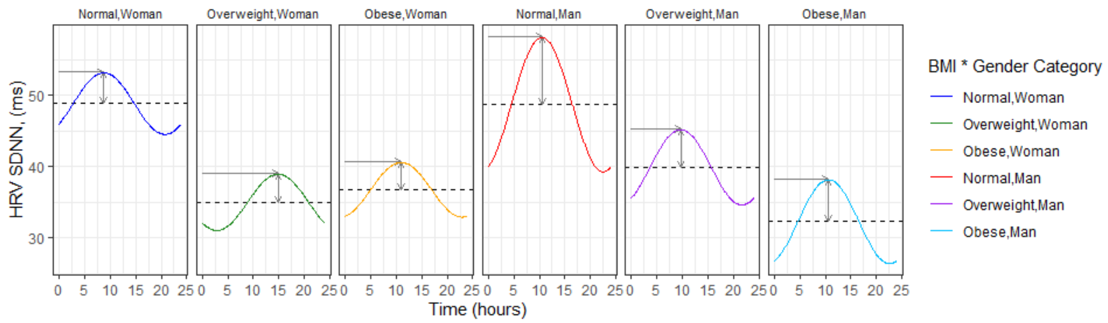

Citation

Please cite:
